# Building the Business Case for an Inclusive Approach to Digital Health Measurement With a Web App (Market Opportunity Calculator): Instrument Development Study

**DOI:** 10.2196/45713

**Published:** 2023-07-26

**Authors:** Mitchell Tang, Yashoda Sharma, Jennifer C Goldsack, Ariel Dora Stern

**Affiliations:** 1 Harvard Business School Harvard University Boston, MA United States; 2 Digital Medicine Society Boston, MA United States; 3 Harvard-MIT Center for Regulatory Science Harvard University Boston, MA United States

**Keywords:** inclusion, digital health, digital product development, health equity, public health, Digital Health Measurement Collaborative Community, DATAcc

## Abstract

**Background:**

The use of digital health measurement tools has grown substantially in recent years. However, there are concerns that the promised benefits from these products will not be shared equitably. Underserved populations, such as those with lower education and income, racial and ethnic minorities, and those with disabilities, may find such tools poorly suited for their needs. Because underserved populations shoulder a disproportionate share of the US disease burden, they also represent a substantial share of digital health companies’ target markets. Incorporating inclusive principles into the product development process can help ensure that the resulting tools are broadly accessible and effective. In this context, inclusivity not only maximizes societal benefit but also leads to greater commercial success.

**Objective:**

A critical element in fostering inclusive product development is building the business case for why it is worthwhile. The Digital Health Measurement Collaborative Community (DATAcc) Market Opportunity Calculator was developed as an open-access resource to enable digital health measurement product developers to build a business case for incorporating inclusive practices into their research and development processes.

**Methods:**

The DATAcc Market Opportunity Calculator combines data on population demographics and disease prevalence and health status from the US Census Bureau and the US Centers for Disease Control and Prevention (CDC). Together, these data are used to calculate the share of US adults with specific conditions (eg, diabetes) falling into various population segments along key “inclusion vectors” (eg, race and ethnicity).

**Results:**

A free and open resource, the DATAcc Market Opportunity Calculator can be accessed from the DATAcc website. Users first select the target health condition addressed by their product, and then an inclusion vector to segment the patient population. The calculator displays each segment as a share of the overall US adult population and its share specifically among adults with the target condition, quantifying the importance of underserved patient segments to the target market. The calculator also estimates the value of improvements to product inclusivity by modeling the downstream impact on the accessible market size. For example, simplifying prompts on a hypertension-focused product to make it more accessible for adults with lower educational attainment is shown by the calculator to increase the target market by 2 million people and the total addressable market opportunity by US $200 million.

**Conclusions:**

Digital health measurement is still in its infancy. Now is the time to establish a precedent for inclusive product development to maximize societal benefit and build sustainable commercial returns. The Market Opportunity Calculator can help build the business case for “why”—showing how inclusivity can translate to financial opportunity. Once the decision has been made to pursue inclusive design, other components of the broader DATAcc toolkit for inclusive product development can support the “how.”

## Introduction

### Background

Digital technologies are playing an increasing role in health care delivery. Digital health measurement tools represent a key technology class at the center of this movement. These tools use connected sensor technologies to collect physiological measurements and other health information during patients’ daily lives. The resulting measurements offer the potential to change how we define health and treat disease. More frequent measurements outside traditional health care settings can help shift care to more patient-centered models and facilitate earlier diagnosis and better access to care. More diverse, continuous, and high-resolution data can also enable a richer understanding of the impacts of medical products—facilitating faster development of better products. Recently, we have seen rapid growth in the use of digital health measurement tools in both care delivery [1,2] and clinical research and development (R&D) [3].

However, there are well-founded concerns that the benefits of digital health measurement will not be shared equitably [4]. Critically, individuals with the highest unmet need—those who are very sick and those for whom traditional care models have proven inadequate—may find these tools inaccessible or inappropriately designed for their needs [5,6]. Digital health has seen large investments over recent years [7], but there remains a risk that aspirations to fast growth and near-term profits may leave historically underserved groups behind. The existing inequities in health care systems have been well documented, and the COVID-19 pandemic has highlighted many downstream consequences of those inequities [8,9]. Yet, digital tools promise greater access and flexibility, rendering them a promising means to mitigate inequities rather than exacerbate them. As we continue to pursue R&D for new digital health products and measurement tools, health equity must be a central focus.

Against this backdrop, the Digital Medicine Society (DiMe) [10] in collaboration with the Food and Drug Administration’s Center for Devices and Radiological Health [11] has launched the Digital Health Measurement Collaborative Community (DATAcc) to create a “toolkit” of best practices for enabling inclusivity throughout the digital health measurement product development process. As a Center for Devices and Radiological Health strategic priority, collaborative communities convene diverse stakeholders across the medical device ecosystem in precompetitive work to achieve common outcomes, solve shared challenges, and leverage collective opportunities [12].

### Introducing the Market Opportunity Calculator

A critical element in fostering inclusive product development is building the business case for why it is worthwhile. While early-stage R&D often happens in nonprofit and academic settings, product development and commercialization are ultimately led by businesses and therefore operate with a focus on commercial benefit. However, the goal of commercial profit is often highly compatible with inclusive product development. Underserved populations, such as racial and ethnic minorities and individuals with disabilities, represent a substantial portion of the US population and therefore a large group of potential beneficiaries of new health tools [13]. In addition, given their higher rates of chronic illness and lower levels of self-reported health, these populations also shoulder a disproportionate share of the US disease burden [14]. In this context, digital health measurement products that are well-suited for these groups will be the only way to reach the total addressable product market. We set out to build a tool to show the value of inclusive digital health measurement technologies. This led to the creation of the DATAcc Market Opportunity Calculator for inclusive development [15].

## Methods

### Data Sources

The data underlying the DATAcc Market Opportunity Calculator were drawn from publicly available sources. The US Census’s 2017 National Population Projections were used to determine the relative size of population segments across key “inclusion vectors,” such as age groups, race and ethnicity, and levels of educational attainment [13]. Data from the 2019 US Census American Community Survey similarly provided statistics on the size of population segments across household income groups [16]. These data sets were combined with data on disease prevalence and health status from the 2018 Centers for Disease Control and Prevention (CDC) National Health Information Survey [14]. The 2019 CDC Disability and Health Data System provided statistics on the size of disability group segments as well as disease prevalence and health status data for those same groups [17]. Segment sizes were represented as percent shares of the sampled population. To calculate the total market size (in terms of number of potential patients), we multiplied shares by an estimate of the current US population from the US Census Population Clock [18].

### Ethical Considerations

Institutional Review Board review was not necessary because our analysis relied solely on publicly available summary statistics from the US Census Bureau and the CDC.

### Data Processing

Calculations from the Market Opportunity Calculator are specifically for the US adult population (ie, those older than 18 years, representing roughly 77% of the total US population) due to the US focus of our key data sources. Age group share data were adjusted to reflect the US adult population. Household income data were incorporated using the assumption that “family” households have 2 adults, while nonfamily households have 1. Educational attainment data are for US adults aged 25 years and older. We applied the 25 and over segment sizes to the full adult population under the assumption that they are likely to be representative of the final educational attainment of the full adult population. Finally, race and ethnicity segment sizes were extrapolated based on the entire US population under the assumption that segment shares for adults were roughly consistent.

For each inclusion vector, we defined mutually exclusive, collectively exhaustive segments. For example, we combined race and ethnicity into a single dimension by creating a separate segment for all individuals of Hispanic or Latino ethnicity regardless of race. The remaining non-Hispanic or Latino population was then segmented by race. Additional details on data processing are provided in Multimedia Appendix 1.

### Analysis

The goal of the opportunity calculator is not only to report segment sizes and rates of disease prevalence within them but also to quantify the importance of often-underserved patient segments to a given market and, by direct extension, the value of increasing a product’s inclusivity for that group. For example, using statistics on the percent of visually impaired individuals with diabetes and the overall prevalence of diabetes, we can determine the percent of diabetes patients that are visually impaired—a useful statistic in quantifying the importance of expanding the inclusivity of a diabetes digital measurement tool to those who are visually impaired.

## Results

### DATAcc Market Opportunity Calculator Overview

**Figure 1:**
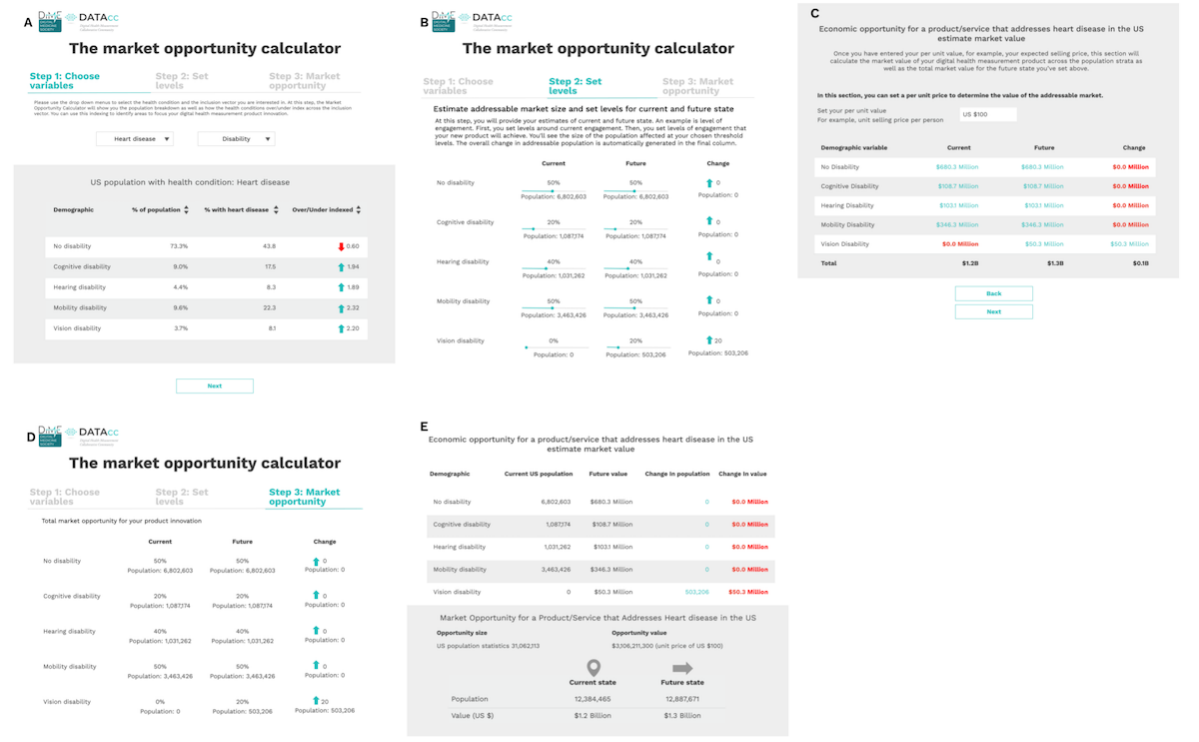
The Digital Health Measurement Collaborative Community (DATAcc) Market Opportunity Calculator, which quantifies the value of increasing the inclusivity of a digital health measurement product. (A) Users start by selecting the target health condition, their product addresses, and an inclusion vector along which to segment the population. The calculator displays each segment as a share of the overall US adult population, its share specifically among adults with the target condition, and the degree to which the segment is over- or underindexed among those with the target condition. (B) The user sets a baseline reflecting the product’s current inclusivity for each segment and projects values for a future state where improvements are achieved. The calculator then shows how these improvements manifest in expansions to the product’s accessible market. (C) When provided with a unit value per product, the calculator quantifies the economic opportunity associated with the market expansion in dollars. (D) Screenshot of the final readout, which shows growth in market size associated with the projected increases in product inclusivity. (E) Screenshot of the final readout showing the market expansion in dollars. Higher-resolution version of this figure is available in Multimedia Appendix 2.

The DATAcc Market Opportunity Calculator (Figure 1) is a freely available, open-access resource designed for use by those doing R&D in digital health measurement to build a case for incorporating inclusive practices in their product development process. The tool can be accessed from the DATAcc website [15].

### Measuring the Importance of Underserved Patient Segments

DATAcc Market Opportunity Calculator users begin by selecting the target health condition their product is intended to address. Current options include arthritis, diabetes, heart disease, high blood pressure, stroke, and fair or poor health status. Next, the user selects an “inclusion vector” along which to segment the population. Current options include age, race and ethnicity, education, household income, and disability. The calculator then displays each segment as a share of the overall US adult population and its share specifically among adults with the target condition (Figure 1A). The calculator output also presents information on which segments are over- or underindexed. For example, while only 3.7% (9.5 million individuals) of the overall US adult population are visually impaired; 8.1% (2.5 million individuals) of those with heart disease are. Thus, among heart disease patients, individuals with vision disabilities are overrepresented by a factor of 2.2.

### Quantifying the Benefits of Improving Product Inclusivity

The calculator also enables users to estimate the value of improvements to their products’ inclusivity for specific segments along the chosen inclusion vector. The user first sets a baseline reflecting the user’s understanding of the product’s current inclusivity for each segment (represented as a percentage of the segment the product is accessible and effective for). They then can consider alternative scenarios in a future state where improvements in inclusivity are achieved (Figure 1B). The calculator shows how these improvements manifest in expansions of the product’s accessible market. By setting a unit price or margin for their product, the user can also quantify the economic opportunity associated with the market expansion in dollars (Figure 1C). Consider the example above of a heart disease-focused digital measurement tool where a user explores improving usability for the visually impaired: if the product could serve 20%, rather than 0%, of visually impaired adults with heart disease, that would represent 500,000 more potential patients. With a per unit margin of US $100, such an improvement would expand the addressable market opportunity by US $50 million.

### Putting it All Together

After these straightforward steps are completed by users of the calculator, a final readout is provided, which shows the potential value of expanding the inclusivity of the focal product, presents additional growth in market size (Figure 1D), and an associated increase in dollar value (Figure 1E). Users of the market opportunity calculator have the option to generate a pdf of the calculations. No data on user input are stored or collected by the calculator.

What users will observe through the use of the calculator is that across disease areas and inclusion vectors there are substantial economic opportunities from increasing inclusivity, whether those be simplifying prompts on a hypertension-focused product to make it more accessible for adults with lower educational attainment, providing Spanish translations to improve use of a diabetes-focused product for the Hispanic and Latino population, or enabling compatibility of an arthritis-focused product with older phone models and low bandwidth connections to make it more accessible to low-income adults (Table 1).

**Table 1:** Inclusivity improvement scenarios modeled with the Digital Health Measurement Collaborative Community (DATAcc) Market Opportunity Calculator^a^.

Improvement scenario	Target condition	Inclusion vector	Segment	Segment size versus total US adult population, %	Segment size versus all adults with target condition, %	Over- or underindexing factor	Gains from accessing 20% more of the target segment	
							Market expansion, patients (in millions), n	Associated economic opportunity (assuming US $100 unit margin)
Improving the usability of a heart disease-focused product for the visually impaired	Heart disease	Disability	Vision disability	3.7	8.1	2.20	0.5	US $50 million
Simplifying prompts on a hypertension-focused product to make it more accessible for adults with lower educational attainment	High blood pressure	Education	Less than high school graduate	11.4	14.2	1.24	2	US $200 million
Providing Spanish translations to improve use of a diabetes-focused product for the Hispanic and Latino population	Diabetes	Race and ethnicity	Hispanic or Latino	17.8	18.7	1.05	1	US $100 million
Enabling compatibility of an arthritis-focused product with older phone models and low bandwidth connections to make it more accessible to low-income adults	Arthritis	Household income	Less than US $35,000	26.5	33.2	1.25	4	US $400 million

^a^Results from 4 potential improvement scenarios that can be modeled using the DATAcc Market Opportunity Calculator. Figures for “market expansion” and “associated economic opportunity” reflect a scenario where improvements allow the product to reach 20% more of the target segment (eg, serving 20% rather than 0% of the target segment) and the product has a US $100 unit margin.

## Discussion

### Principal Findings

The Market Opportunity Calculator, along with other components of the DATAcc toolkit for inclusivity, supports the implementation of an inclusive approach to digital health measurement product development [15]. Lack of financial incentives is a major barrier to achieving health equity, so creating a business case for inclusive product design is imperative. The Market Opportunity Calculator demonstrates that inclusive product development can result in expanded market size and financial opportunity, providing the necessary incentives for digital health product developers—and their investors—to adopt an inclusive approach.

Results from the Market Opportunity Calculator can be paired with the Library of Evidence Supporting Inclusive Design, also included in the DATAcc toolkit, which highlights specific case studies providing concrete evidence of the benefits of inclusive design, including broadening product usage, improving outcomes, and reducing risk [15]. Once a decision is made to continue on the path of inclusivity, the digital health measurement product development process, modeled after the Food and Drug Administration’s device development process, outlines the key steps in the product development lifecycle where inclusivity elements can be applied. The Framework for Inclusive Development walks through each step and provides recommendations and resources to apply these inclusivity elements. At present, numerous companies, such as AliveCor [19], Verily [20], and Genentech [21], have begun to leverage the Market Opportunity Calculator and other components of the DATAcc toolkit to articulate the value of inclusivity and better incorporate inclusive design principles into their development processes. Additionally, DiMe recently shared the calculator as a resource for the diversity, equity, and inclusion in Digitized Clinical Trials project [22]. The calculator demonstrates how real-world data can be used to identify additional populations who may benefit from inclusion in clinical trials.

In recent years, several studies in the academic literature and popular press have highlighted instances where lack of inclusive design in medical products have resulted in care inequities: pulse oximeters that are inaccurate for certain skin tones [6] and telehealth apps that are inaccessible to the visually impaired [23]. Identifying these issues is the first step toward remedying them. However, ultimately, the goal should be proactive inclusivity, rather than reactive inclusivity. We will only realize the full promise of the digitization of health care if inclusivity is woven into the initial development of the product itself, rather than something to be fixed after the fact. However, as shown by the DATAcc Framework for Inclusive Development, inclusive design takes time and resources—to consult with or hire accessibility experts, recruit diverse populations for usability testing, and implement the requisite features. The Market Opportunity Calculator shows that the investment in inclusive digital product design is worth it, not just from a moral or equity lens but from a business case perspective as well.

### Limitations

In this paper, we describe the development of and data from the first iteration of the calculator, which has a number of limitations. First, the calculator currently includes a limited number of inclusion vectors and health conditions. The limited number of health conditions in particular may constrain the use of the tool for companies developing products for conditions outside the selection set. However, in these instances, developers may still use the general “fair or poor rated health” category as a rough proxy for the target condition and still glean insight on the relative importance of historically underserved patient segments. Second, users currently can only select a single inclusion vector at a time, whereas the selection of multiple vectors may enable more granular population segmentation. Third, the data underlying the model were collected from 2017 to 2019; however, we believe these statistics were unlikely to change dramatically over the recent years. Finally, the calculator only provides data for the US adult population.

DiMe, through work by the DATAcc, will update the calculator as additional input data become available. Future iterations will hope to incorporate more recent data, include additional health conditions and inclusion vectors, and allow for the selection of multiple inclusion vectors simultaneously. The target for the next calculator release is sometime in the first half of 2024.

### Conclusions

Digital health measurement has grown rapidly over recent years but is still in its infancy. Now is the time to lay a foundation ensuring that inclusivity is woven into the fabric of the industry. Doing so will set the industry up for long-term success: aligning business incentives with the unmet needs of underserved populations and delivering both sustainable commercial profits and maximizing the societal benefits created by digital measurement tools.

## Appendix

Multimedia Appendix 1 Additional details on data processing for the Digital Health Measurement Collaborative Community (DATAcc) market opportunity calculator.

Multimedia Appendix 2 The Digital Health Measurement Collaborative Community (DATAcc) Market Opportunity Calculator, which quantifies the value of increasing the inclusivity of a digital health measurement product. (A) Users start by selecting the target health condition, their product addresses, and an inclusion vector along which to segment the population. The calculator displays each segment as a share of the overall US adult population, its share specifically among adults with the target condition, and the degree to which the segment is over- or underindexed among those with the target condition. (B) The user sets a baseline reflecting the product’s current inclusivity for each segment and projects values for a future state where improvements are achieved. The calculator then shows how these improvements manifest in expansions to the product’s accessible market. (C) When provided with a unit value per product, the calculator quantifies the economic opportunity associated with the market expansion in dollars. (D) Screenshot of the final readout, which shows growth in market size associated with the projected increases in product inclusivity. (E) Screenshot of the final readout showing the market expansion in dollars. Higher resolution version of Figure 1.

## Abbreviations

CDC: US Centers for Disease Control and Prevention
DATAcc: Digital Health Measurement Collaborative Community
DiMe: Digital Medicine Society
R&D: research and development

## Data Availability

All data generated or analyzed during this study are included in this published article and its supplementary information files.

## References

[1] Tang M, Nakamoto CH, Stern AD, Mehrotra A (2022). Trends in remote patient monitoring use in traditional medicare. JAMA Intern Med.

[2] Tang M, Mehrotra A, Stern AD (2022). Rapid growth of remote patient monitoring is driven by a small number of primary care providers. Health Aff (Millwood).

[3] Marra C, Chen JL, Coravos A, Stern AD (2020). Quantifying the use of connected digital products in clinical research. NPJ Digit Med.

[4] Ramsetty A, Adams C (2020). Impact of the digital divide in the age of COVID-19. J Am Med Inform Assoc.

[5] Thies K, Anderson D, Cramer B (2017). Lack of adoption of a mobile app to support patient self-management of diabetes and hypertension in a federally qualified health center: interview analysis of staff and patients in a failed randomized trial. JMIR Hum Factors.

[6] Sjoding MW, Dickson RP, Iwashyna TJ, Gay SE, Valley TS (2020). Racial bias in pulse oximetry measurement. N Engl J Med.

[7] Landi H (2022). Digital health startups banked record-breaking $29.1B last year. Will the momentum continue in 2022?. Fierce Healthcare.

[8] Ndugga N, Artiga S (2021). Disparities in health and health care: 5 key questions and answers. Kaiser Family Foundation.

[9] Mishra V, Seyedzenouzi G, Almohtadi A, Chowdhury T, Khashkhusha A, Axiaq A, Wong WYE, Harky A (2021). Health inequalities during COVID-19 and their effects on morbidity and mortality. J Healthc Leadersh.

[10] DiMe—advancing digital medicine to optimize human health. Digital Medicine Society.

[11] (2022). Center for devices and radiological health. US Food and Drug Administration.

[12] (2021). Collaborative communities: addressing health care challenges together. US Food and Drug Administration.

[13] (2017). 2017 national population projections tables: main series. United States Census Bureau.

[14] (2018). National health interview survey: tables of summary health statistics. US Centers for Disease Control and Prevention.

[15] DATAcc inclusivity: toolkit for digital health measurement product development. Digital Medicine Society.

[16] (2019). American community survey (ACS). United States Census Bureau.

[17] (2019). Disability and health data system (DHDS). US Centers for Disease Control and Prevention.

[18] U.S. and world population clock. United States Census Bureau.

[19] (2022). AliveCor case study. DATAcc.

[20] (2022). Verily case study. DATAcc.

[21] (2022). Genentech case study. DATAcc.

[22] (2023). Diversity, equity, and inclusion in digitized clinical trials. Digital Medicine Society.

[23] Alexiou G (2022). Inaccessible telehealth apps don’t just exclude – they’re a matter of life and death. Forbes.

