# Supplementary material for: Building the Business Case for an Inclusive Approach to Digital Health Measurement With a Web App (Market Opportunity Calculator): Instrument Development Study
Source: JMIR Form Res. 2023 Jul 26;7:e45713. doi: 10.2196/45713 (PMC10413230; doi:10.2196/45713)
Supplement: Multimedia Appendix 1 [file formative_v7i1e45713_app1.docx]

**Data sources**

Five data sources supply inputs into the DATAcc Market Opportunity Calculator

- **Source A:** US Census’s 2017 National Population Projections (link: <https://www.census.gov/data/tables/2017/demo/popproj/2017-summary-tables.html>)
- **Source B:** 2018 CDC National Health Information Survey (link: <https://www.cdc.gov/nchs/nhis/shs/tables.htm>)
- **Source C:** 2019 US Census American Community Survey (link: <https://data.census.gov/cedsci/>)
- **Source D:** 2019 CDC Disability and Health Data System (link: <https://www.cdc.gov/ncbddd/disabilityandhealth/dhds/index.html?CDC_AA_refVal=https%3A%2F%2Fwww.cdc.gov%2Fncbddd%2Fdisabilityandhealth%2Fdhds.html>)
- **Source E:** US Census population clock (link: <https://www.census.gov/popclock/>)

Inputs from these sources can be organized into three categories: (1) segment size data (population demographics), (2) disease prevalence and health status data, and (3) overall market size data.

**1. Segment size data**

Segment sizes were drawn from sources A, C, and D. Additional details on specific calculations are included below, organized by inclusion vector.

*Age*

Age segment sizes were drawn from source A. These data reflect the US population as a whole; however, we adjusted that data to focus only on the adult population. We defined 3 broad age segments: "18-44", "45-64", and "65+". In our model, segment sizes must be linked to disease prevalence and health status data. These three segments match the least granular age segmentations in the health data. When more granularity was provided, we aggregated to these three segments.

In the US Census data, 22.7% of the population are under 18 years of age. Because we were interested only in the remaining 77.2%, we scaled each segment in the original data up by 100/77.2 to determine segment sizes as a percent of the US adult population.

*Race & ethnicity*

Race & ethnicity segment sizes were drawn from source A, which includes race & ethnicity data for the entire US population (including non-adults). By applying these segment sizes, we assumed that segment sizes for adults were not meaningfully different from those for the population as a whole.

US census data represent race & ethnicity as two distinct dimensions. Along the race dimension, individuals can be identified as having one race (either "White”, “Black or African American”, “American Indian and Alaska Native”, “Asian”, and “Native Hawaiian / Other Pacific Islander”) or two or more races. Additionally, regardless of race, individuals can be classified based on ethnicity as either Hispanic/Latino or non-Hispanic/Latino.

For the purposes of our calculator, we collapsed the separate race and ethnicity dimensions into a single mutually exclusive, collectively exhaustive segmentation. We created a separate segment for all individuals of "Hispanic or Latino" ethnicity regardless of race. The remaining non-Hispanic/Latino population was broken down by race. "Native Hawaiian and Other Pacific Islander" was the smallest of these segments (0.18% of the population). Because our disease prevalence and health status data sources do not provide information on this segment, we excluded them from the analysis. We scaled all other segments up by 100/99.8 to ensure that the remaining segment sizes summed to 100%.

*Education*

Education segment sizes were drawn from source C. These data provide estimates of the educational attainment for the US population over age 25. We combined the highest two education levels "Bachelor's degree" and "Graduate or Professional degree" into a single category, "Bachelor's degree and above", to be consistent with our health data.

Oftentimes final educational attainment is used in social science as a proxy for other factors (e.g., upbringing) that might influence outcomes like health or technology adoption. Therefore final educational attainment is more relevant than educational attainment at the present. We applied the 25 and over education segment sizes to the full adult population with the assumption that they were roughly representative of the final educational attainment for the full adult population.

*Household income*

Household income data were drawn from source C. We aggregated the more granular source data into 5 segments: "Less than $35,000", "$35,000 to $49,999", "$50,000 to $74,999", "$75,000 to $99,999", and "$100,000 or more". We converted household level data to individual level data by assuming that family households have 2 adults, while non-family households only have 1.

*Disability*

Disability prevalence data were drawn from source D. Though data from the source is age-adjusted to 2000 US age demographics, we applied them to our model under the assumption that age demographic changes since 2000 did not materially affect disability prevalence rates. Statistics were provided for four types of disability that could be linked to our disease prevalence and health status data.: "Cognitive Disability", "Hearing Disability", "Mobility Disability", and "Vision Disability”.

Our 4 disability groups are not mutually exclusive; individuals can have multiple disabilities. The data show that 73.3% of the US adult population have none of our 4 examined disabilities, leaving 26.7% with at least one. However, summing across our 4 disability groups, we arrived at 35.7% of the population, revealing a degree of double counting. To obtain a mutually exclusive, collectively exhaustive segmentation, we allocated the 26.7% disability share across the 4 disability groups based on their prevalence in the population.

**2. Disease prevalence and health status data**

Disease prevalence and health status data were pulled for five health conditions: "Fair or poor rated health", "Heart disease", "High blood pressure", "Stroke", “Diabetes”, and "Arthritis". Additional notes are included below, organized by inclusion vectors.

*Age, race & ethnicity, education, and household income*

Unadjusted disease prevalence and health status data for age, race & ethnicity, education, and household income were provided from source B. For age disease prevalence estimates, we combined the "65-74 years" and "75 years and over" segments into a single "65+" segment based on a weighted average using age segment sizes from source A. For race & ethnicity estimates, non-Hispanic/Latino health data were provided for the "White" and "Black or African American" segments only. For the remaining non-Hispanic race segments we used the overall race prevalence rates instead (i.e., including both Hispanic and non-Hispanic individuals of that race).

*Disability*

Health condition prevalence data for disability segments were drawn from source D. Similar to the disability segment size data, these figures were age-adjusted to 2000 US age demographics. We applied them under the assumption that age demographic changes since 2000 did not materially affect health condition prevalence rates.

**3. Market size data**

Overall market sizes for each health condition were calculated in terms of number of adults. We started with the total US population drawn from the US Census Population Clock (accessed on 2/8/22). This figure is updated every second, but changes are small and do not materially impact our market sizes. We multiplied this by 77.2%, the share of the US population that are adults, to determine the total US adult population. We then calculated the market size for each health condition by multiplying its overall prevalence by the total US adult population. Overall health condition prevalence rates were drawn from Source B.
