# Supplementary figures and images for: Building the Business Case for an Inclusive Approach to Digital Health Measurement With a Web App (Market Opportunity Calculator): Instrument Development Study
Source: JMIR Form Res. 2023 Jul 26;7:e45713. doi: 10.2196/45713 (PMC10413230; doi:10.2196/45713)

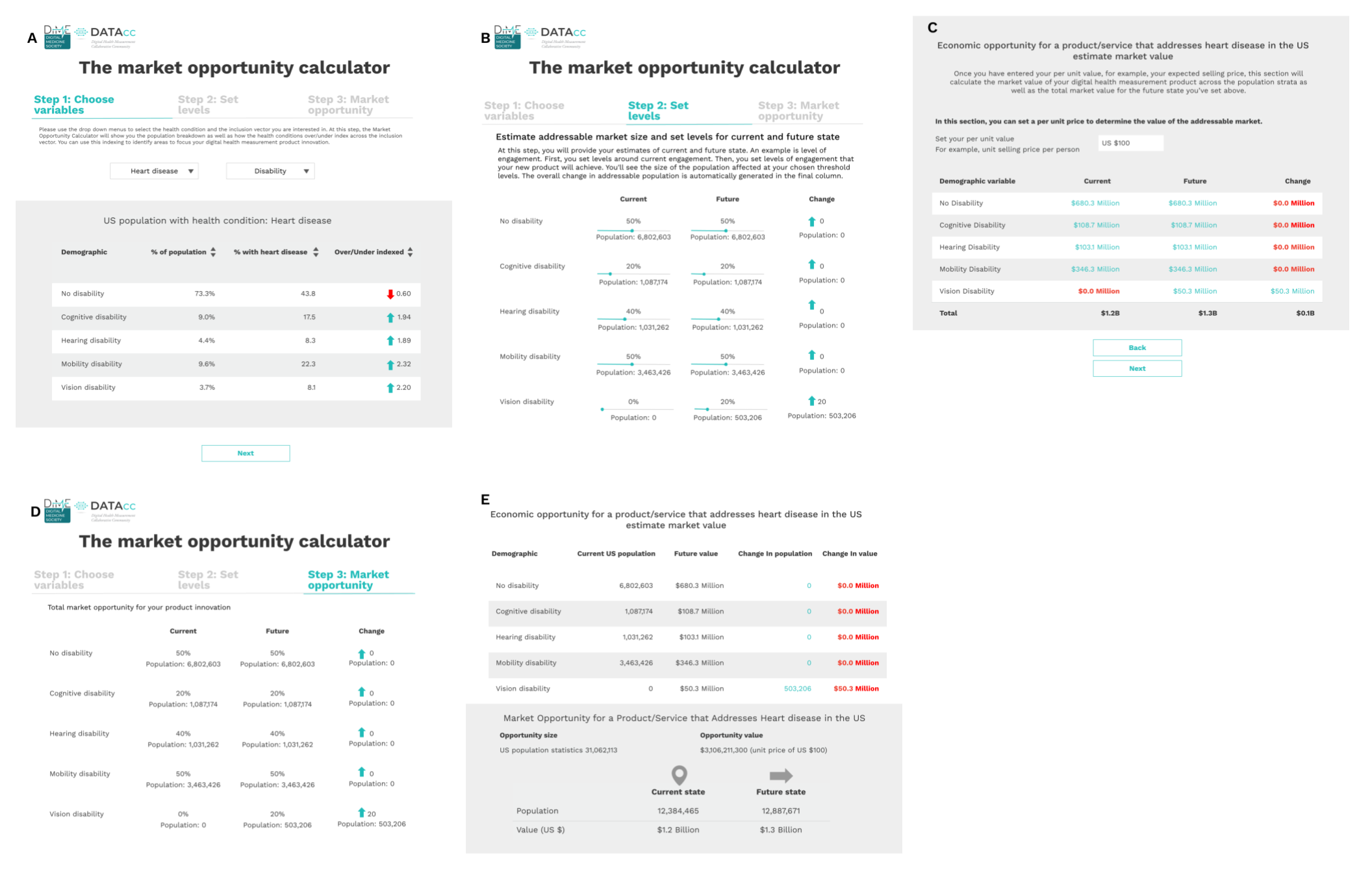

Supplement: Multimedia Appendix 2 [file formative_v7i1e45713_app2.png]
